# Subcortical Correlates of Individual Differences in Aptitude

**DOI:** 10.1371/journal.pone.0089425

**Published:** 2014-02-25

**Authors:** Rex E. Jung, Sephira G. Ryman, Andrei A. Vakhtin, Jessica Carrasco, Chris Wertz, Ranee A. Flores

**Affiliations:** 1 Department of Neurosurgery, University of New Mexico, Albuquerque, New Mexico, United States of America; 2 Department of Psychology, University of New Mexico, Albuquerque, New Mexico, United States of America; Brainnetome Center, & National Laboratory of Pattern Recognition, China

## Abstract

The study of individual differences encompasses broad constructs including intelligence, creativity, and personality. However, substantially less research is devoted to the study of specific aptitudes in spite of their importance to educational, occupational, and avocational success. We sought to determine subcortical brain structural correlates of several broad aptitudes including Math, Vocabulary, Foresight, Paper Folding, and Inductive Reasoning in a large (N = 107), healthy, young (age range  = 16–29) cohort. Subcortical volumes were measured using an automated technique (FreeSurfer) across structures including bilateral caudate, putamen, globus pallidus, thalamus, nucleus accumbens, hippocampus, amygdala, and five equal regions of the corpus callosum. We found that performance on measures of each aptitude was predicted by different subcortical structures: Math – higher right nucleus accumbens volume; Vocabulary – higher left hippocampus volume; Paper Folding – higher right thalamus volume; Foresight – *lower* right thalamus and higher mid anterior corpus callosum volume; Inductive Reasoning – higher mid anterior corpus callosum volume. Our results support general findings, within the cognitive neurosciences, showing lateralization of structure-function relationships, as well as more specific relationships between individual structures (e.g., left hippocampus) and functions relevant to particular aptitudes (e.g., Vocabulary).

## Introduction

Substantial research has been conducted relating individual differences across measures of intelligence, creativity, and personality to various brain measures of structure and function [1]–[3]. Such research is undertaken to better understand individual behavioral characteristics that might predict performance across major adaptive life tasks including employment, relationships, and avocational pursuits [4]–[6]. Rather understudied have been the relationships between subcortical structures and measures of higher cognitive functioning, although such relationships are emerging across measures of intelligence [7], creativity [8], and personality [9].

Despite their well-established relationship with educational [10] and occupational success [11], specific aptitudes have been studied substantially less than broad constructs relevant to individual differences. Indeed, more precise measures of ability focused on specific aptitudes provide highly useful information to individuals, particularly relevant to job choice [12], [13]. Of the few aptitudes studied, musical and math aptitudes have been associated most commonly with variation in brain structure and function. For example, one study found that mathematically gifted students activated a unique network of brain regions when solving a 3-dimensional mental rotation task when compared with subjects of average math ability [14]. Similarly, both musical aptitude and pitch perception in musicians has been well associated with morphological and neurophysiological changes within Heschl's gyrus [15], [16].

While the gray and white matter correlates of such measures of individual differences are of keen interest to the neuroscience community, the relative contribution of major subcortical structures (hippocampus, amygdala, caudate, thalamus, etc.) remains relatively understudied in spite of the relative importance of these structures to most major neurological and psychiatric disorders of behavior. For example, the role of the hippocampus in episodic memory was highlighted by the unfortunate case of H.M. (Henry Molaison), who suffered severe memory deficits following excision of bilateral mesial temporal lobe structures to cure his epilepsy [17]. A common finding in patients diagnosed with schizophrenia, effectively treated with antipsychotic medications, is increased volume of the caudate nucleus [18]. Lesions to the dorsomedial nucleus of the thalamus have been associated with executive dysfunction in neurological patients [19]. A recent neurological conceptualization links many of these subcortical structures together in “circuits;” the function (or dysfunction) of which can recapitulate higher cortical function central to cognitive, mood, and motor functioning [20]. Thus, in such exploratory analyses of individual differences (such as aptitude), we believe that it is appropriate to begin with brain structures that represent more primary components underlying cognitive functioning, with very well established brain-behavior correlates having been documented in the extant literature.

We sought to determine the role of several subcortical structures in individual differences relevant to four measures of aptitude used in vocational guidance (i.e., Vocabulary, Foresight, Paper Folding, Inductive Reasoning), and one measure widely used in broader psychological studies (i.e., mathematical ability). This approach allows, for the first time, the integrity of subcortical structures central to higher cognitive functioning to be related to individual differences across a wide range of aptitudes relevant to educational, occupational, and avocational pursuits. There exists nearly universal support for the notion that greater volume of such subcortical structures is associated with better behavioral performance, and lower volume (or lesion) is associated with behavioral impairment or disruption [21], although studies in individual differences in creativity also show occasional inverse relationships see [22] for review. Thus, we hypothesize that subcortical structure volumes would be associated with better performance across all measures of aptitude.

## Methods

This study was conducted according to the principles expressed in the Declaration of Helsinki. The study was approved by the Institutional Review Board of the University of New Mexico (IRB#11-531). All subjects provided written informed consent before collection of samples and subsequent data analysis.

### Subjects and Procedure

One hundred and seven subjects (60 males, 47 females) between the ages of 16 and 29 (Mean  = 20, s.d.,  = 2.8) were included in the study. All subjects were screened by questionnaire and were free from neurological (e.g., epilepsy, traumatic brain injury) and psychiatric (e.g., major depressive) disorder. Subjects were excluded if they described using recreational drugs (e.g., cocaine, ecstasy, etc.); however, alcohol and marijuana use was allowed provided marijuana was not used in the previous 24 hours. All participants were administered a 3 hour battery including tests of intelligence, creativity, personality, and aptitude, for which they were compensated $20 per hour.

### Imaging Acquisition and Processing

**Figure 1:**
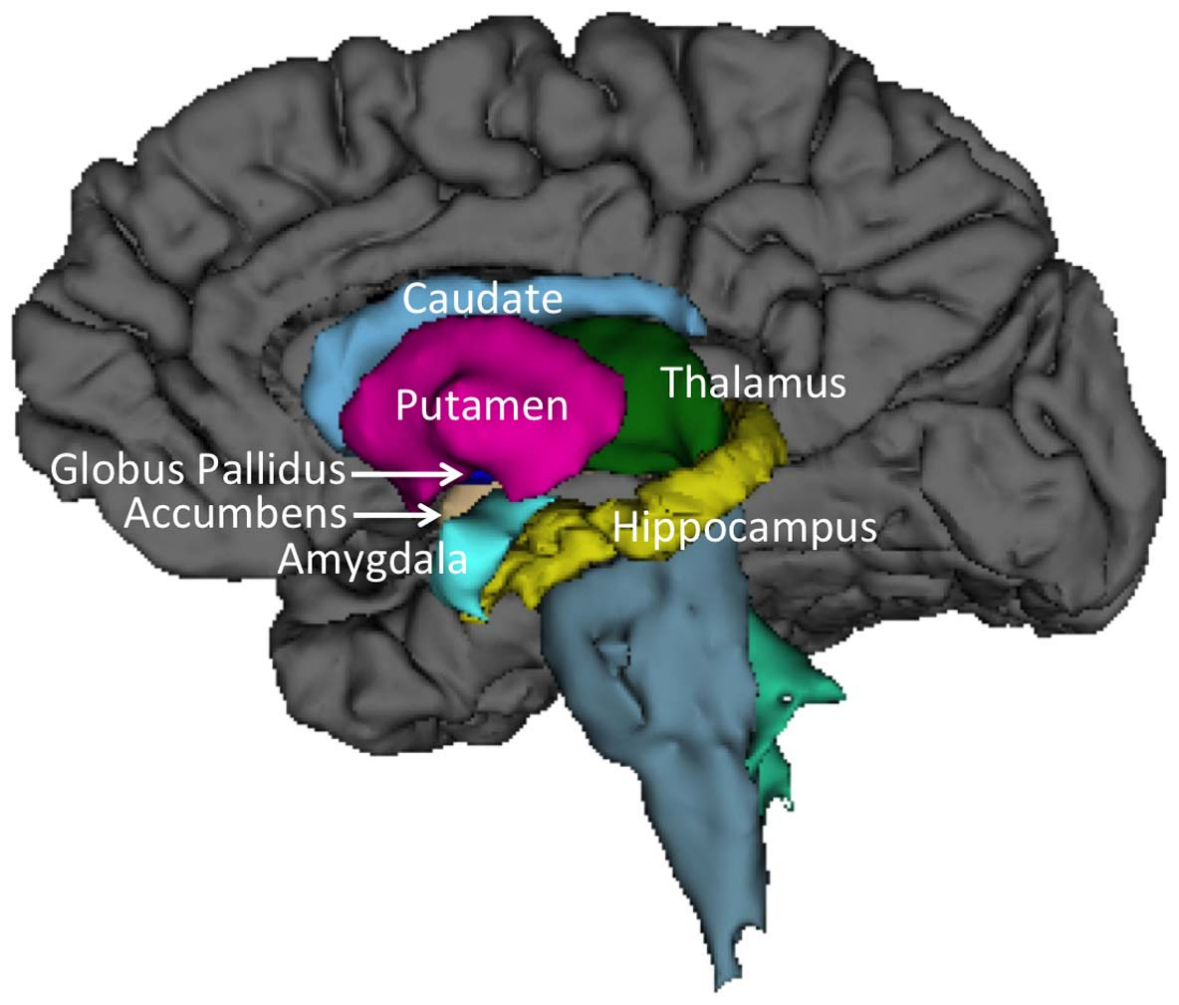
Sagittal view of subcortical structures with segmentation examples from FreeSurfer of Caudate (light blue), Putamen (hot pink), Thalamus (green), Globus Pallidus (dark blue), Nucleus Accumbens (light brown), Amygdala (turquoise), and Hippocampus (yellow).

Structural imaging was obtained on a 3 Tesla Siemens scanner using a 32-channel head coil to obtain a T1 5 echo sagittal MPRAGE sequence [TE = 1.64 ms; 3.5 ms; 5.36 ms; 7.22 ms; 9.08 ms; TR = 2530 ms; voxel size  = 1.0×1.0×1.0 mm^3^; FOV = 256 mm; slices  = 192; acquisition time  = 6:03]. Scans were reviewed for image quality. Cortical reconstruction and volumetric segmentation were performed with the FreeSurfer image analysis suite (http://surfer.nmr.mgh.harvard.edu/) described in several papers [23]–[25]. For this study, we focused on the sub-cortical volume segmentation results including the caudate, putamen, globus pallidus, thalamus, nucleus accumbens, hippocampus, amygdala, and corpus callosum (Figure 1). Measures of the corpus callosum were automatically segmented into five regions including: anterior, mid-anterior, central, mid-posterior, and posterior.

### Aptitude Tests

All subjects were administered four tests in the Johnson O'Connor Research Foundation battery including: Vocabulary, Foresight, Paper Folding, and Inductive Reasoning. The Johnson O'Connor battery of tests was developed in the 1920's to assist General Electric in matching employees to particular job demands, with the Foundation later emerging to provide career guidance. Several abilities were identified, and the test battery has been refined to include 19 ability tests ranging from Memory for Designs to Pitch Discrimination. Validities and reliabilities of all measures have been determined across thousands of subjects, and available via technical reports, with reliabilities (and discriminant validity) for all measures being recently reported [26]. The Johnson O'Connor tests have been recently used as measures of individual differences in several neuroimaging studies [27]–[29]. Vocabulary (reliability  = .96) measures subjects' knowledge of English words – a crystallized measure of verbal facility; subjects are given one target word, and are asked to choose one from four possible words that is closest in meaning to the target word. Foresight (reliability  = .96) measures subjects' divergent thinking ability – a measure of creative capacity; subjects are presented with a design and asked to write as many things that the design reminds them of. Paper Folding (reliability  = .82) measures the ability to visualize three-dimensional forms – a measure of structural visualization ability; subjects mentally visualize a piece of paper as it is folded, punched with a paper punch, and unfolded. Inductive reasoning (reliability  = .84) measures the ability and quickness in seeing a common element among facts, ideas, and observations – a measure of induction; subjects are given six pictures, quickly identifying the three pictures that go together. Subjects were also administered one of two versions of the Graduate Record Examination Math test, provided by Educational Testing Services, under a standard 40-minute time limit. Only 99 of the 107 subjects completed the Math GRE due to addition of this test to the battery at a slightly later time-point.

### Statistical Analysis

Pearson correlation coefficients were used to assess relationships between aptitude measures. To examine the relationships between regional volumes and aptitude scores, stepwise linear regression was performed. All subcortical volumes were entered in a stepwise manner (i.e., as independent variables), predicting total score of each aptitude measure (i.e., Vocabulary, Foresight, Paper Folding, Inductive Reasoning, Math) as dependent variables, while controlling for age and sex (entered in step one of the regression equation). Raw values obtained from FreeSurfer output were used. Statistical threshold was set at p = .05 for each of the independent regressions for this exploratory study with planned contrasts involving *a priori* hypotheses, to properly balance the potential for Type I and Type II error [30], [31]. We used Akaike Information Criterion (AIC) with standard modeling for variable selection purposes due to multicollinearity of subcortical measures. Selected variables (Independent Variables) were entered into a stepwise linear regression to predict each aptitude measure (Dependent Variable), controlling for age and sex (Entered in Step 1). We used IBM SPSS version 20.0 for Mac for all statistical analyses.

## Results

Scores on each aptitude measure were as follows: Math (Range 1–23, Mean  = 12.26, s.d.  = 4.74); Vocabulary (Range 3–22, Mean  = 10.9, s.d.  = 4.4); Paper Folding (Range 0–72, Mean  = 29.7, s.d.  = 15.2); Foresight (Range 23–103, Mean  = 52.1, s.d.  = 15.0); Inductive Reasoning (Range 79–181, Mean  = 130.7, s.d.  = 24.3). Vocabulary was significantly correlated with Math ability (r = .36, p<.001), and Paper Folding was significantly correlated with Math ability (r = .50, p<.001). No other significant correlations were observed among measures of aptitude.

Many subcortical measures were significantly correlated, reflecting multicollinearity of such measures. Controlling for age and sex, within structures, left and right bilateral structures were invariably correlated with one another (e.g., caudate – r = .90, putamen – r = .76, globus pallidus – r = .52, nucleus accumbens – r = .59, thalamus – r = .61, hippocampus – r = .59, amygdala – r = .67). Other significant subcortical correlations (Bonferroni correction  = .05/19 = .003) are presented in Table 1.

**Table 1:** Bivariate correlation coefficients between subcortical structures and significance** at p<.003 (Bonferroni correction at .05/19).

	CC Ant	CC Ant/Mid	CC Mid	CC Post/Mid	CC Post	L Caudate	L Putamen	L Pallidum	L Accumb	L Thalamus	L Hippo	L Amygdala	R Caudate	R Putamen	R Pallidum	R Accumb	R Thalamus	R Hippo	R Amygdala
CC Ant	1																		
CC Ant/Mid	0.27	1																	
CC Mid	0.27	.82**	1																
CC Post/Mid	.40**	.55**	.60**	1															
CC Post	.69**	.36**	.28**	.49**	1														
L Caudate	.44**	0.07	−0.05	0.21	.49**	1													
L Putamen	.20	−0.02	−0.11	0.14	0.16	.45**	1												
L Pallidum	.17	−0.1	−0.22	−0.08	0.19	.42**	.47**	1											
L Accumb	−.10	−0.03	−0.05	0.02	−0.13	−0.02	0.27	0.14	1										
L Thalamus	.40**	.28**	.35**	.32**	.50**	0.21	0.06	−0.16	−.30**	1									
L Hippo	.23	0.19	0.26	0.15	0.13	0.26	.31**	0.15	.02	.31**	1								
L Amygdala	.00	0.02	−0.05	−0.1	−0.05	0.12	.30**	0.38**	.35**	−.06	.20	1							
R Caudate	.48**	0.01	−0.12	0.11	.51**	.90**	.41**	.46**	−.01	.22	.25	.14	1						
R Putamen	.16	0.08	−0.06	0.16	0.15	.41**	.76**	.52**	.14	.16	.28	.30**	.42**	1					
R Pallidum	.42**	0.21	0.06	0.27	.50**	.58**	.50**	.52**	−.06	.37**	.24	.20	.59**	.52**	1				
R Accumb	−.05	−0.03	−0.08	0.17	−0.04	0.16	.41**	.31**	.59**	−.31**	−.01	.36**	.11	.34**	.09	1			
R Thalamus	.38**	0.17	0.25	0.13	.35**	0.23	0.2	.30**	−.11	.61**	.43**	.21	.25	.22	.31**	−.06	1		
R Hippo	.26	0.24	0.21	0.06	0.12	.29**	0.21	.27	.05	.17	.59**	.27	.28	.35**	.20	.05	.40**	1	
R Amygdala	.03	0	−0.1	−0.1	−0.02	0.27	.37**	.36**	.41**	−.13	.31**	.67**	.30**	.36**	.22	.38**	.13	.37**	1.00

L – left hemisphere structure; R – right hemisphere structure; CC – Corpus Callosum; Ant – Anterior; Ant/Mid – Anterior/Midbody; Mid – Midbody; Post/Mid – Posterior/Midbody; Post – Posterior; Pallidum – Globus Pallidus; Accumb – Nucleus Accumbens; Hippo – Hippocampus.

AIC variable selection and predictor importance for each aptitude measure were as follows: Math – right thalamus (.36), left amygdala (.29), right nucleus accumbens (.20), right amygdala (.15); Vocabulary – right putamen (.34), left caudate (.21), left hippocampus (.18), left putamen (.16), right caudate (.11); Paper Folding – right thalamus (.29), right caudate (.24), CC anterior (.22), right nucleus accumbens (.14), right globus pallidus (.11); Foresight – right thalamus (.72), CC mid anterior (.28); Inductive Reasoning – left nucleus accumbens (.38), left thalamus (.32), CC mid anterior (.30).

**Figure 2:**
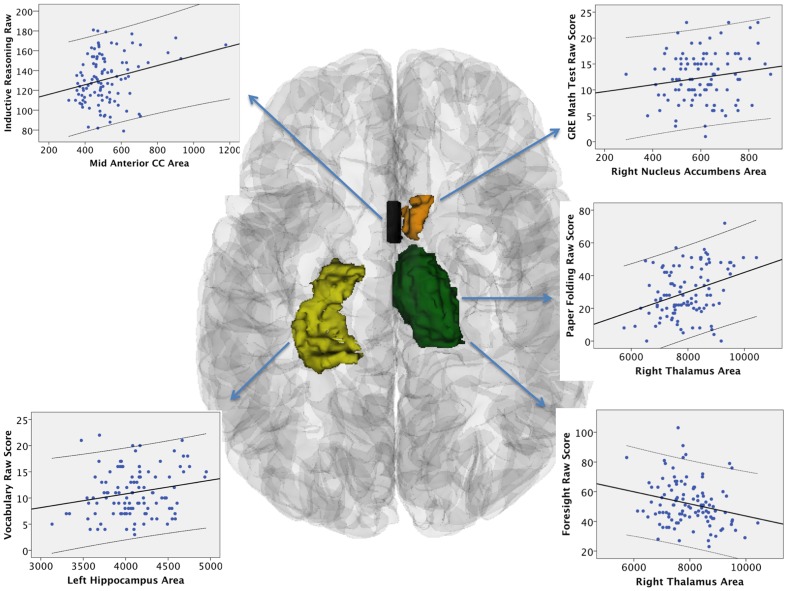
Axial view of subcortical structures related to aptitude measures, with scatter plots displaying linear relationships (solid line) and 95% confidence intervals (dashed lines) for all subjects. Four structures are shown: Hippocampus (yellow), Mid Anterior Corpus Callosum (black), Nucleus Accumbens (brown), Thalamus (green).

Finally, we tested the linear relationship between each of the five aptitude measures and subcortical volume measures (selected by AIC), controlling for age and sex. Math was predicted by higher right nucleus accumbens volume (F = 3.9, p = .01, Adjusted R^2^ = .08, Beta = .22). Vocabulary was predicted by higher left hippocampus volume (F = 3.2, p = .03, Adjusted R^2^ = .06, Beta = .22). Paper Folding was predicted by higher right thalamus volume (F = 5.5, p = .001, Adjusted R^2^ = .11, Beta = .31). Foresight was predicted by a model including *lower* right thalamus and higher mid anterior corpus callosum volume (F = 2.7, p = .03, Adjusted R^2^ = .06, Beta thalamus  = −.27, Beta CC = .21). Inductive Reasoning was predicted by higher mid anterior corpus callosum volume (F = 2.9, p = .04, Adjusted R^2^ = .05, Beta = .25). Figure 2 shows bivariate scatterplots for each measure of aptitude (Y axis) as compared to each subcortical structure (X axis).

## Discussion

Our results demonstrate significant, differential subcortical contributions to the expression of various aptitudes in a large young sample. These subcortical relationships were directional in that higher volumes were associated with better performance across measures of aptitude, save for Foresight in which an inverse relationship was found. Both left and right lateralized structure-function relationships were observed, with broadly consistent left lateralized relationships being observed for verbal tasks (i.e., Vocabulary-left hippocampus) and right lateralized relationships observed on a predominantly non-verbal task (i.e., Paper Folding-right thalamus). Thus, the results generally conform to broad lateralization of brain-behavior relationships observed within the cognitive neurosciences [32].

More specifically, we found compelling relationships between specific subcortical structures and corresponding aptitudes. For example, the relationship observed between Vocabulary-left hippocampus integrity is well supported by both clinical and non-clinical research highlighting the importance of the left hippocampus in semantic processes including: learning a novel lexicon [33], processing of novel versus familiar words [34], and semantic memory retrieval [35]. The measure of Foresight, most analogous to measures of divergent thinking used in creativity studies, was also compelling given previous findings. Foresight was inversely related to the right thalamus volume, and positively related to mid anterior corpus callosum volume. Our previous research has found inverse relationships between the integrity of white matter of the anterior thalamic radiation and creative cognition as measured by standard measures of divergent thinking [36], and other researchers have demonstrated relationships between the integrity of mid and anterior aspects of the corpus callosum and measures of divergent thinking [37]. In a recent review, we noted several findings showing inverse relationships between measures of brain structural integrity (whether measured by volume, fractional anisotropy, or lesion) and creative cognition [22], a preponderance of these findings being within regions identified as the default mode network [38]. The current findings add to the hypothesis, articulated in Jung et al., of a disinhibitory network of brain regions associated with increased behavioral output (Page 3).”

Several other relationships were observed between subcortical structures and aptitudes, which are less well supported by the extant literature, but are equally compelling. Math aptitude was predicted by right nucleus accumbens volume – a relationship supported by research showing relationships between activation of the right nucleus accumbens (and caudate) to mediate the relationship between anxiety and performance on math tests [39]. The nucleus accumbens, more broadly, is a structure central to motivated behavior [40] and regulation of effortful functioning [41], two broad behavioral features that would appear important to performance on measures of high-level math. The relationship between Paper Folding and right thalamic volume is rather non-specific, although lateralized lesions of the right thalamus have been found to result in visuo-spatial deficits [42]. Finally, the relationship between Inductive Reasoning and the anterior corpus callosum is rather non-specific, although again, one would expect reasoning processes to be broadly mediated by bilateral frontal lobe regions, connected via anterior corpus callosum structures [1].

Aptitudes are generally considered to be inherited, although scientific support for this notion is generally limited. Our results do not suggest whether environmental or genetic factors predominate in structure-function relationships, given the cross sectional design. The results would indicate that structural correlates of important aptitudes relevant to verbal, creative, visuo-spatial, math, and inductive reasoning abilities are detectable at early ages (16–29) at which individuals are attempting to match their individual skills to educational, occupational, and avocational goals. Moreover, the behavioral tests appear to be tapping into individual differences that are manifested in the structure of the human brain. Future research will be necessary to disentangle the genetic versus environmental contribution of such structure-function relationships and the extent to which these aptitudes may be modified through intense environmental influence is possible, if at all [43].

There are several limitations of this study. First, we focused only upon subcortical structures of the brain, as opposed to the entire brain, in looking at structure-function relationships of various aptitudes. As this is an exploratory study, we felt that such an approach was warranted given the enormous number of possible relationships that might exist between gray and white matter regions and our subset of aptitudes. The possibility of Type I error is increased when such exploratory studies encompass the entire brain. Thus, we felt that a reasonable approach was to start by looking at subcortical structures, which have very well-defined structure-function relationships and which are rather limited in number. With the current findings, we can progress to white matter and gray matter inquiries using the current results as hypothesis generators as opposed to open-ended enquiries encompassing the entire brain. Second, we studied only young, healthy subjects, limiting the generalizability of the findings. Future studies should determine whether our results extend across different age and demographic populations (e.g., psychiatric, neurological populations), although the current findings are most relevant to individuals seeking aptitude testing. Finally, the measures are few, given that aptitude testing can encompass several hours of testing, with measures of very finite abilities (e.g., Color Discrimination). Our approach was to sample broadly from aptitudes that would be relevant to many educational, occupational, and avocational interests (e.g., word knowledge, creativity, visuo-spatial, math, inductive reasoning). More finite abilities might result in very different results, although this possibility must await future studies.

We sought to determine the possible relationship between the volume of several subcortical structures and five broad measures of aptitude. We found that larger volume of several structures corresponded to better performance of most aptitudes, except for a measure of creative cognition where results conformed to findings of inverse structure-function relationships. Our results conformed, generally, to well-established findings within the cognitive neurosciences showing lateralization of structure-function relationships (e.g., Vocabulary-left hippocampus; Paper Folding-right thalamus). The findings also were supported by individual findings within the neurosciences showing more specific relationships between specific structures (e.g., left hippocampus) and functions relevant to particular aptitudes (e.g., Vocabulary). Future studies will be undertaken to extend these results into white- and gray matter correlates, with targeted hypotheses based on cortical loops linking subcortical structures in cohesive networks.
